# Attenuation of Krüppel-Like Factor 4 Facilitates Carcinogenesis by Inducing G1/S Phase Arrest in Clear Cell Renal Cell Carcinoma

**DOI:** 10.1371/journal.pone.0067758

**Published:** 2013-07-05

**Authors:** Erlin Song, Xin Ma, Hongzhao Li, Peng Zhang, Dong Ni, Weihao Chen, Yu Gao, Yang Fan, Haigang Pang, Taoping Shi, Qiang Ding, Baojun Wang, Yu Zhang, Xu Zhang

**Affiliations:** 1 Department of Urology/State Key Laboratory of Kidney Diseases, Chinese PLA General Hospital/Medical School of Chinese PLA, Beijing, China; 2 Department of Urology, Chinese PLA 211 Hospital, Harbin, China; Philipps University, Germany

## Abstract

Krüppel-like factor 4 (KLF4) is a transcription factor with diverse functions in various cancer types; however, the function of KLF4 in clear cell renal cell carcinoma (ccRCC) carcinogenesis remains unknown. In this study, we initially examined KLF4 expression by using a cohort of surgically removed ccRCC specimens and cell lines. Results indicated that the transcription and translation of KLF4 were lower in ccRCC tissues than in patient-matched normal tissues. Furthermore, the KLF4 expression was significantly downregulated in the five ccRCC cell lines at protein and mRNA levels compared with that in normal renal proximal tubular epithelial cell lines (HKC). KLF4 downregulation was significantly correlated with tumor stage and tumor diameter. Promoter hypermethylation may contribute to its low expression. In addition, *in vitro* studies indicated that the KLF4 overexpression significantly inhibited proliferation in human ccRCC cell lines 786-O and ACHN. Moreover, the KLF4 overexpression arrested the cell cycle progress at the G1/S phase transition by upregulating p21*^WAF1/CIP1^* expression and downregulating cyclin D1 expression, KLF4 knockdown in HKC cells did the opposite. *In vivo* studies confirmed the anti-proliferative effect of KLF4. Our results suggested that KLF4 had an important function in suppressing the growth of ccRCC.

## Introduction

Renal cell carcinoma (RCC) ranks second among the leading causes of deaths in patients with urologic tumors and accounts for 2% of adult malignancies [1]. In patients who suffer from RCC, clear cell RCC (ccRCC) comprises approximately 80% of the histological subtype [2]. Radical or partial nephrectomy is one of the most effective treatments for ccRCCs; however, the prognosis is extremely poor for advanced and metastatic ccRCCs, which are resistant to chemotherapy and radiotherapy. In ccRCC, the von Hippel-Lindau gene (VHL) alteration is common,drugs that modulate the downstream targets of pVHL/HIF and PI3K/AKT/mTOR pathways have been used for ccRCC treatment. However, advanced or metastatic ccRCCs remain untreated because these drugs have various limitations, including inability to relieve all patients [3] and the lack of a stable drug efficacy biomarker [4]. To develop effective diagnostic, preventive, and treatment methods for ccRCCs, further studies on the pathogenesis of ccRCC are needed.

In our previous work, a profile was performed by using human primary ccRCC and metastatic ccRCC tissues. Compared to primary ccRCC, the expression of KLF4 in metastatic ccRCC was reduced 67%. Then, a series of experiments were conducted to verify the role of KLF4 in ccRCC.

Krüppel-like factor 4 (KLF4), also known as Gut-enriched Krüppel-like factor or epithelial zinc finger, is a member of the Krüppel-like transcription factor family [5], [6]. KLF4, which is highly expressed in differentiated epithelial cells, regulates diverse cellular processes, including cell proliferation, differentiation, and maintenance of normal tissue homeostasis. KLF4 is also involved in cancer stem cell or stem cell renewal [7]–[11].

Evidence has indicated that KLF4 can bind directly to gene promoter regions, which participate in regulating cell cycle progression, such as p21*^WAF1/CIP1^* [12], p27*^Kip1^* [13], [14], cyclin D1 [15], and cyclin B [16]. Thus, KLF4 triggers cell cycle checkpoints to prevent inappropriate cell cycle progression. The degradation of KLF4 eliminates cell cycle inhibitory effects, thereby allowing the cells to reenter the cell cycle. Therefore, KLF4 can regulate the cell cycle progression by controlling G1/S or G2/M boundaries of the cell cycle.

KLF4 functions as a tumor suppressor in several tissues in various cancers, such as colorectal [17], prostate [18], hepatic [19], gastric [20], and intestinal cancers [21]. KLF4 also has an important function in the development of primary lung cancer. For instance, KLF4 expression is decreased in most primary lung tumors compared with case-matched normal lung tissues, and ectopic KLF4 expression significantly inhibits cell growth [22]. Upregulation of KLF4 in the breast and the skin leads to breast cancer [23] and squamous cell carcinoma [24], respectively. KLF4 expression is also a prognostic indicator for several tumor types, including head and neck squamous cell carcinoma [25], colon cancer [26], prostate cancer [18], and hepatic cancer [19]. However, the cytoplasmic location of KLF4 expression is a poor prognostic factor in early-stage breast cancer [27]. The function of KLF4 in skin tumorigenesis remains unclear. A study has shown that the induced KLF4 expression in basal keratinocytes initiates squamous epithelial dysplasia [28]. On the other hand, another study has revealed a tumor suppressive function of KLF4 in a KLF4/CreER™ mouse model [29].

However, the function and detailed mechanisms of KLF4 in ccRCC development and progression remain unknown. In the present study, we showed for the first time that KLF4 was aberrantly expressed in ccRCC. We also found that KLF4 could be a novel tumor suppressor in the development and/or progression of ccRCC.

## Results

### KLF4 was Inactive in Primary ccRCC Specimens

**Figure 1:**
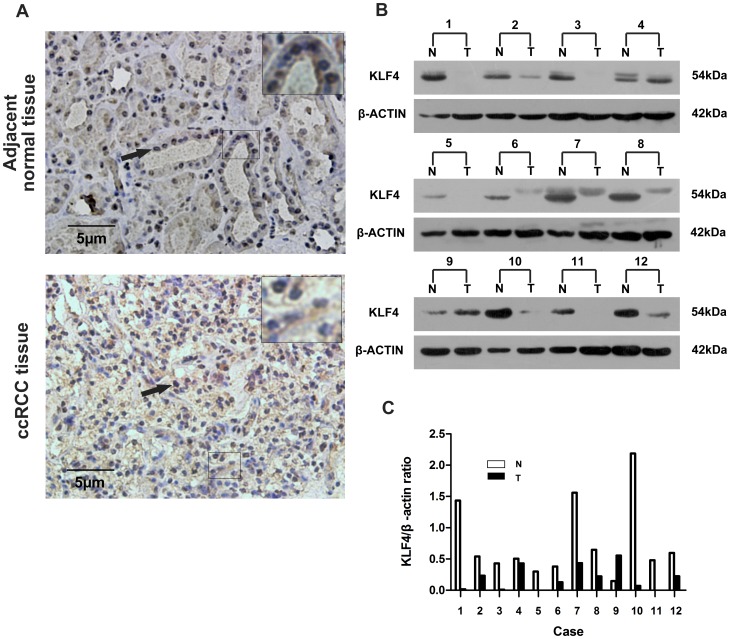
KLF4 protein expression was suppressed in primary ccRCC tissues. (A) Representative immunostaining of KLF4 expression in human ccRCC tissues and corresponding adjacent kidney tissues. Arrows indicate a positive nuclear staining for KLF4. (B) Western blot showing KLF4 expression in ccRCC tissues (T) and corresponding adjacent kidney tissues (N) from ccRCC patients. β-actin is used as a loading control. (C) KLF4 in each specimen was quantified by densitometric analysis using Adobe Photoshop. KLF4 expressions in each sample were normalized with those of β-actin by determining the KLF4:β-actin ratio (K/β-actin). N: normal; T: tumor.

We initially analyzed the KLF4 expression in 41 formalin-fixed, paraffin-embedded human ccRCC specimens and their corresponding adjacent normal tissues. KLF4 localized in the nucleus of all the cells that positively exhibited different expression levels of ccRCC and the normal kidney (Figure 1A). A significant decrease in KLF4 expression was observed in ccRCC tumor tissues compared with that in patient-matched adjacent normal tissues. KLF4 was expressed with “++” or “+++” in the 41 normal tissue samples but with “+” or negative in 25 of 41 tumor samples. However, only 14 primary ccRCC tumors are expressed with “++” and 2 with “+++.” A significant difference in KLF4 expression was observed between the ccRCC tissues and their corresponding adjacent kidney tissues. Table 1 shows the relationship between the KLF4 downregulation in the ccRCC samples and the clinico-pathological parameters of patients with ccRCC. KLF4 downregulation was significantly correlated with tumor stage and tumor diameter (*P*<0.05). However, no significant correlation was found between KLF4 expressions and age, gender, and Fuhrman grade. These results were further confirmed by western blot. KLF4 expression was lower in 21 of 24 (87.5%) ccRCC samples than in adjacent normal tissues (Figure 1B, C). These data indicated that KLF4 may function in ccRCC progress.

**Table 1:** Correlation between KLF4 downregulation and clinico-pathological parameters in patients with ccRCC.

	Total	KLF4 expression level		
Factors	(*n* = 41)	Negative and +	++ and +++	*P*-value
		N(%)	N(%)	
Age (years)				
≤50	16	10(24.4)	6(14.6)	0.8728
>50	25	15(36.6)	10(24.4)	
Gender				
Male	32	19(46.3)	13(31.7)	1.0000
Female	9	6(14.6)	3(7.3)	
Tumor stage				
pT1	25	21(51.2)	4(9.8)	*0.0003
pT2/T3	16	4(9.8)	12(29.2)	
Fuhrman grade				
Grade 1/2	33	20(48.8)	13(31.7)	1.0000
Grade 3/4	8	5(12.2)	3(7.3)	
Tumor diameter (cm)				
≤7	31	15(36.6)	16(39.0)	*0.0032
>7	10	10(24.4)	0(0)	

* Statistically significant.

### KLF4 Promoter is Hypermethylated in Human Primary ccRCC

**Figure 2:**
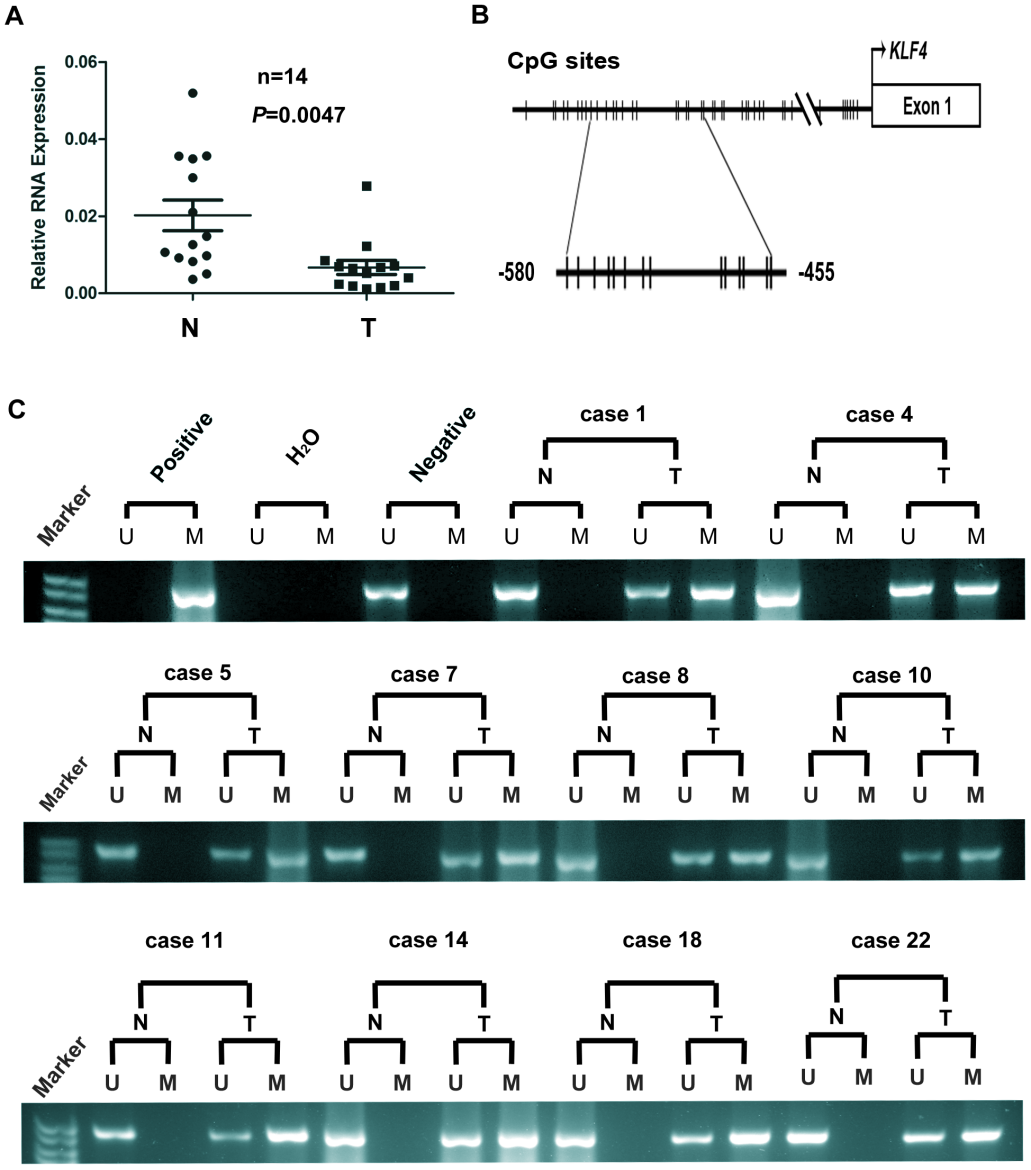
KLF4 was frequently methylated in human primary ccRCC. (A) KLF4 mRNA downregulation in ccRCC tissues. KLF4 expression was quantified by ΔΔCt method, in which PPIA was used as a control sample. N: normal; T: tumor (*P*<0.01). (B). Schematic structure of the KLF4 promoter CpG island. Analysis of exon 1, CpG, transcription start, and MSP sites. (C) MSP analysis of KLF4 methylation in primary ccRCC tissues and normal tissues. Ten cases with a methylated allele are shown. The methylated allele was observed in 11 of 25 (44%) primary ccRCC tissues but was absent in the corresponding normal tissues. Normal blood lymphocyte DNA (NL), in vitro methylated DNA (IVD), and water were used as the negative control treatment, the positive control, and the blank sample, respectively. N: normal; T: tumor; M: methylated; U: unmethylated.

To verify the western blot analysis and immunohistochemistry results, real-time PCR assays were performed. The results showed that KLF4 was expressed at a low level in 12 of 14 (85.7%) ccRCC tissues compared with normal tissues (*P*<0.01; Figure 2A).

Given that the promoter region of KLF4 contains typical CpG islands [19], [22], [30]and hypermethylation is potentially involved in KLF4 transcription regulation, the methylation status of the KLF4 promoter was detected by MSP analysis. A typical CpG island that spans the proximal promoter and exon 1 regions of the KLF4 gene is found at http://cpgislands.usc.edu/ (Figure 2B). The methylated allele was observed in 11 of 25 (44%) primary ccRCC tissues (Figure 2 C) but was not observed in its corresponding normal tissues. This result indicated that the hypermethylated KLF4 promoter region may reduce its transcription.

### KLF4 was Absent and Inhibited the Proliferation in ccRCC Cell Lines

**Figure 3:**
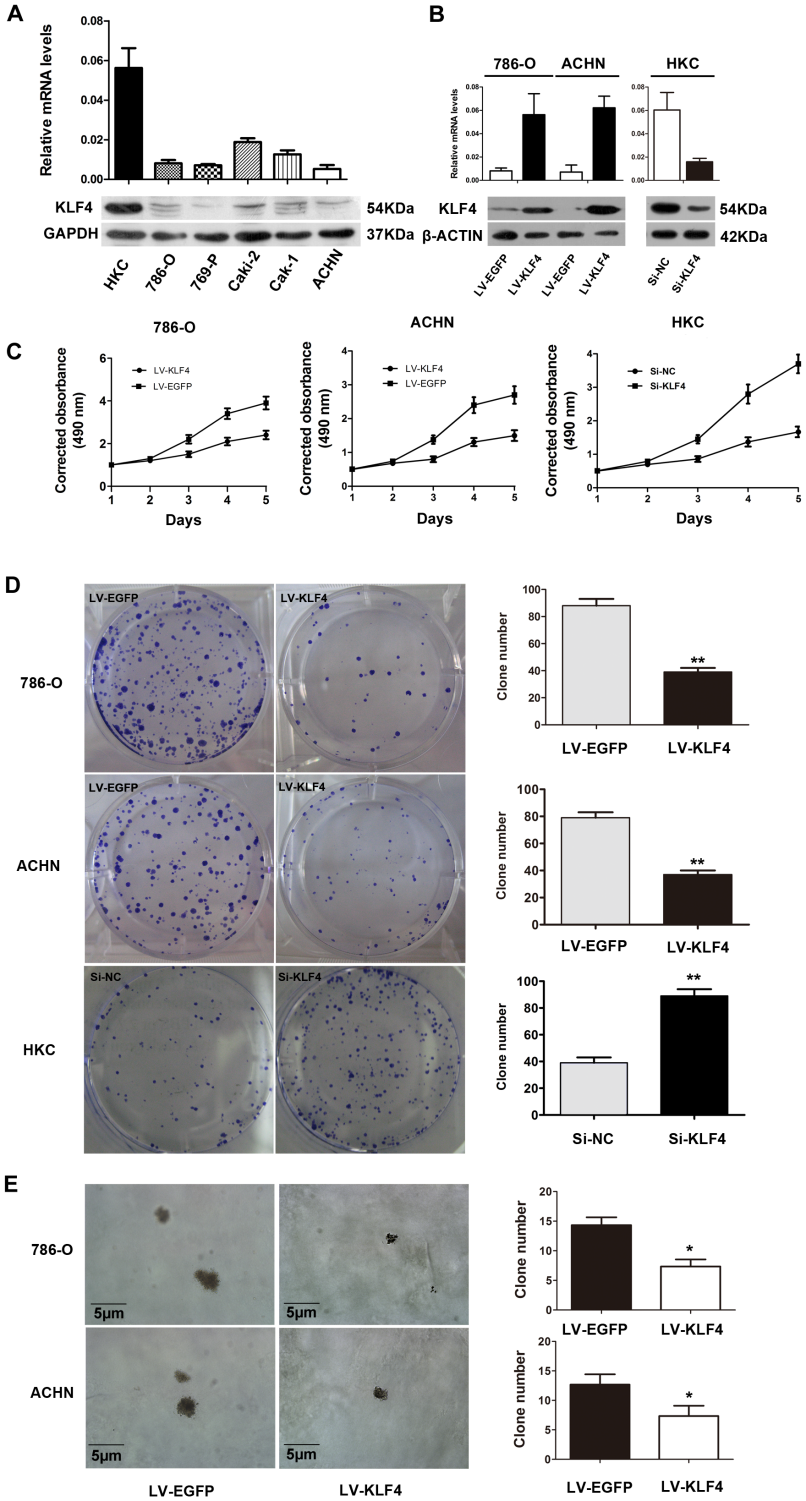
KLF4 was absent and inhibited the proliferation in ccRCC cell lines. (A) KLF4 expression in normal renal proximal tubular epithelial cell line HKC and ccRCC cell lines. KLF4 expression was reduced in the five ccRCC cell lines compared with the HKC cells. Top: KLF4 RNA downregulation in ccRCC cell lines, in which PPIA was used as a control sample. Bottom: KLF4 protein downregulation in ccRCC cell lines, in which GAPDH was used as a control sample. (B) KLF4 expression after 786-O and ACHN cells were infected with LV-EGFP or LV-KLF4 for 96 h. Real-time PCR and western blot analyses were performed to confirm the infection efficiency. The expression levels of KLF4 in HKC cells was also determined after KLF4 knockdown. PPIA and β-actin were used separately as the control treatments. (C) MTS assay revealed the decreased proliferation in 786-O and ACHN cell after KLF4 was overexpressed, the converse effect was also shown after KLF4 knockdown in HKC cells. The data shown are means from two independent experiments and each experiment was performed in triplicate. (D) Induced KLF4 expression significantly inhibited the growth of 786-O and ACHN cells. A opposite effect was observed after KLF4 knowkdown in HKC cells. Left panel: Representative anchorage-dependent growth assay of 786-O, ACHN and HKC cells. Right panel: Quantitative analyses of the colony numbers are shown as mean±SD. The efficiency of anchorage-dependent growth was significantly inhibited (**P<0.01) in KLF4-overexpressed cells compared with that in blank vector-transformed control group in 786-O and ACHN cells, promoted cell growth can be observed after KLF4 knockdown in HKC cells. (E) KLF4 overexpression significantly inhibited the anchorage-independent growth ability in 786-O and ACHN cell lines compared with the control treatment. Left panel: Photomicrographs of the representative fields in which the cells with induced KLF4 expression (LV-KLF4) formed fewer and smaller colonies in the soft agar than those in the vector-transfected (LV-EGFP) cells in 786-O and ACHN cells. Right panel: Quantitative analyses of the colony numbers are shown as mean±SD, *P<0.05 versus the LV-EGFP.

To determine the function of KLF4 in ccRCC cell lines, the KLF4 expression in ccRCC cell lines and normal renal proximal tubular epithelial cell lines (HKC) was examined. The KLF4 expression was reduced in the five ccRCC cell lines at protein and mRNA levels compared with that in the HKC cell (Figure 3A). 786-O and ACHN were transfected with LV-EGFP and LV-KLF4 expression vectors, respectively. MTS and anchorage-dependent growth assays were performed. Figure 3C and D show that the KLF4 overexpression significantly reduced the proliferation of 786-O and ACHN cells, in contrast, knockdown of KLF4 promoted cell proliferation in HKC cells. KLF4 also inhibited the anchorage-independent growth of 786-O and ACHN cells (Figure 3 E). The restoration of KLF4 could also inhibit the migration, invasion and motilities of 786-O cells (File S3, Figure S1 and Figure S2).

### KLF4 Induced Cell Cycle Arrest at G1/S Phase by Modulating p21*^WAF1/CIP1^* and Cyclin D1 Expression in ccRCC Cell Lines

**Figure 4:**
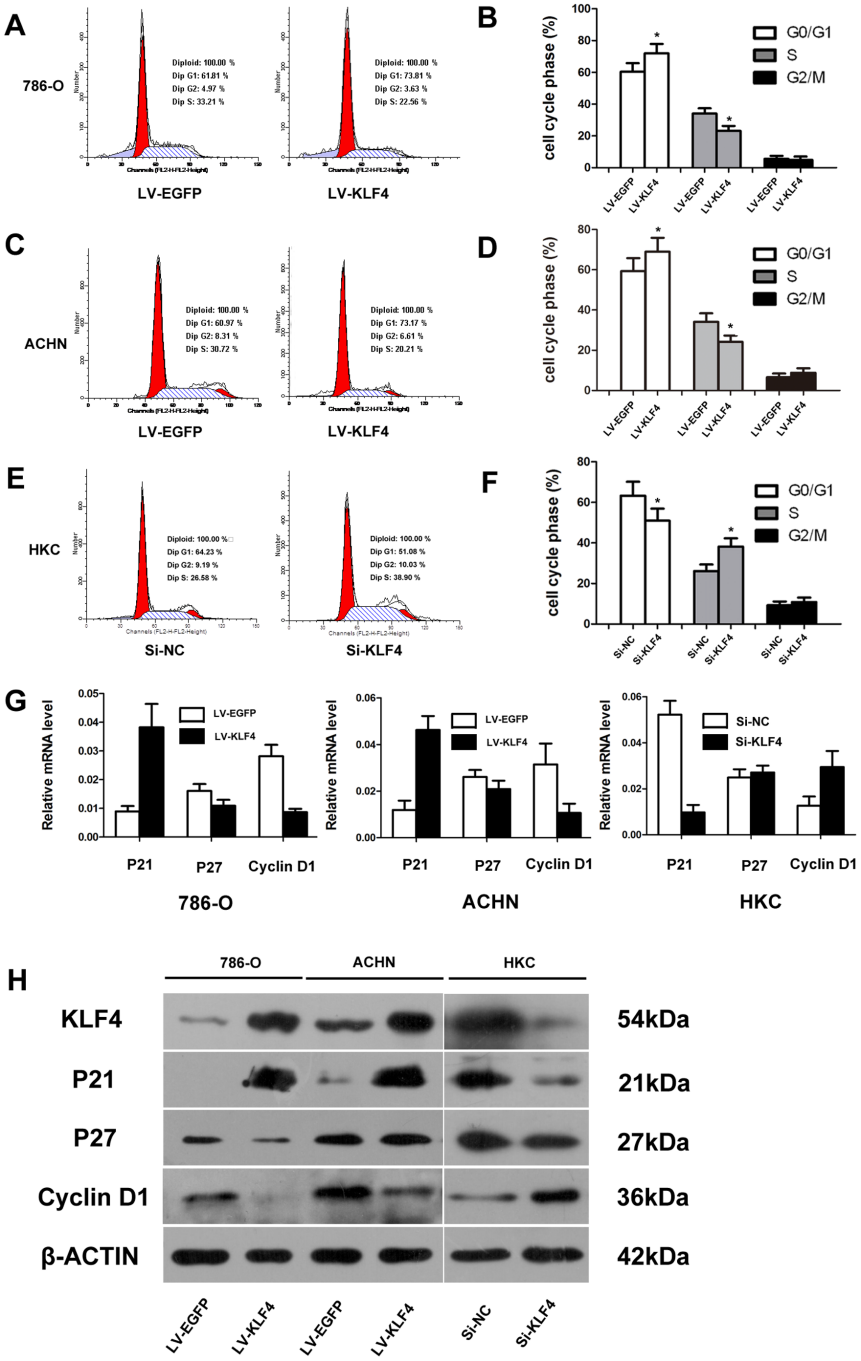
Altered KLF4 expression affected the cell cycle. (A) KLF4 blocked the G1/S phase cell cycle transition of 786-O cells. The percentage of 786-O cells at the G0/G1 phase increased significantly to 73.81% but decreased to 22.56% at the S phase. (B) Quantitative analysis is shown for the cell cycle distribution of LV-EGFP and LV-KLF4 in 786-O cells (*P<0.05 versus the LV-EGFP). (C) KLF4 blocked the G1/S phase cell cycle transition of ACHN cells. The percentage of ACHN cells in G0/G1 phase increased significantly to 73.17% but decreased to 20.21% at the S phase cells. (D) Quantitative analysis is shown for the cell cycle distribution of LV-EGFP and LV-KLF4 in ACHN cells (*P<0.05 versus the LV-EGFP). (E) After KLF4 knockdown, the percentage of HKC cells at the G0/G1 phase significantly decreased from 64.23% to 51.08%, the percentage of cells at S phase increased from 26.58% to 38.90%. (F) Quantitative analysis is shown for the cell cycle distribution of Si-NC and Si-KLF4 in HKC cells (*P<0.05 versus the Si-NC). (G) Real-time PCR results of KLF4, cyclin D1, p21*^WAF1/CIP1^*, and p27*^KIP1^* expressions in 786-O and ACHN cells after KLF4 overexpression, the expression level of KLF4, cyclin D1, p21*^WAF1/CIP1^*, and p*^27KIP1^* in HKC cells was also investigated after KLF4 knockdown. PPIA was used as a loading control. (H) Western blot analysis results of KLF4, cyclin D1, p21*^WAF1/CIP1^*, and p27*^KIP1^* expressions in 786-O and ACHN cells after KLF4 overexpression, western blot analysis was also performed to investigate the expressions of cyclin D1, p21*^WAF1/CIP1^*, and p27*^KIP1^* after KLF4 knockdown. β-actin was used as a loading control.

KLF4 inhibits the proliferation of colon, lung, and cervical carcinoma cell lines by blocking G1/S phase arrest [13], [22], [31]. However, the cell cycle progression is also arrested as induced by KLF4 at the G2/M phase in the prostate cancer cell line [18]. To determine the effect of KLF4 on ccRCC cell cycle, flow cytometry was performed. Figures 4 A and C showed that the percentage of 786-O and ACHN cells at the G0/G1 phase significantly increased to 73.81% and 73.17%, respectively. The percentage of cells at the S phase decreased to 22.56% and 20.21%, respectively. Figures 4 E showed that after KLF4 knockdown, the percentage of HKC cells at the G0/G1 phase significantly decreased from 64.23% to 51.08%, the percentage of cells at S phase increased from 26.58% to 38.90%. These results suggested that KLF4 induced the cell cycle arrest at the G1/S phase transition in ccRCC cell lines.

KLF4 regulates the expression of several G1/S cell cycle-related genes, including cyclin D1, p21*^WAF1/CIP1^*, and p27*^Kip1^* [14], [15], [32]. To determine whether or not KLF4 functions in regulating the expression of these genes in ccRCC, we investigated the expressions of KLF4, cyclin D1, p21*^WAF1/CIP1^*, and p27*^Kip1^* in selected ccRCC cell lines by real-time PCR and western blot analyses. The KLF4 overexpression significantly enhanced the p21*^WAF1/CIP1^* expression and reduced the cyclin D1 expression, this overexpression did not modulate p27*^Kip1^* expression in 786-O and ACHN cells. On the contrary, a converse result was observed after KLF4 knockdown in HKC cells (Figure 4G, H).

### KLF4 Suppressed ccRCC Cell Growth *in vivo*

**Figure 5:**
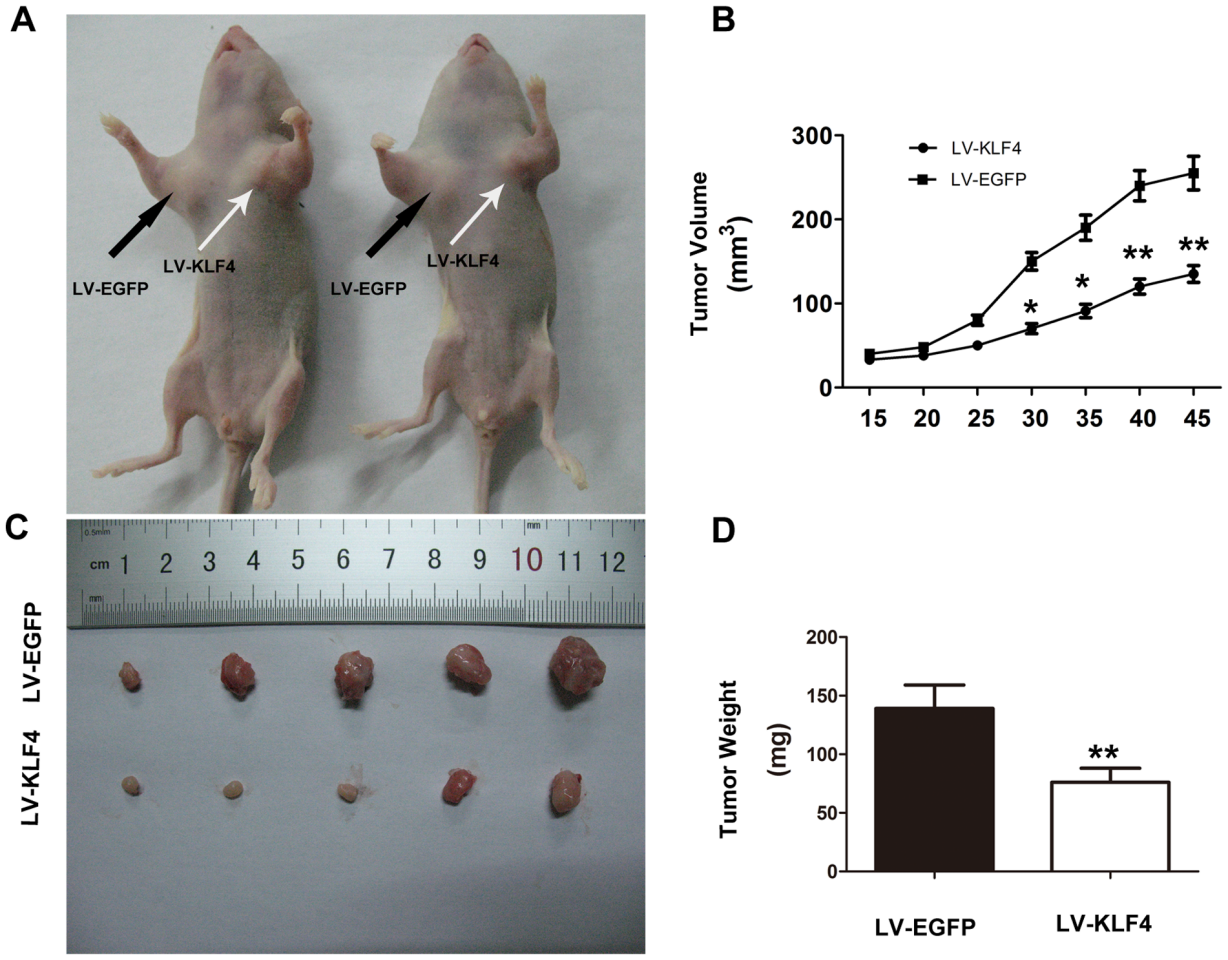
Induced KLF4 expression suppressed tumor growth ***in vivo.*** (A) Representative nude mice showing the different morphologies of the tumors derived from LV-KLF4-transfected 786-O cells (right side) and LV-EGFP-transfected control cells (left side). (B) Tumor growth curve of LV-KLF4- and LV-EGFP-infected 786-O cells in nude mice. Tumor sizes were determined as described in the Materials and Methods (*P<0.05;**P<0.01). (C) Photograph of tumors excised from nude mice 45 days after inoculation of 786-O cells infected with LV-KLF4 (Top) and LV-EGFP (Bottom). (D) The averages of tumor weights derived from the LV-KLF4 group were significantly lower than those from the LV-EGFP group when the nude mice were sacrificed on day 45 after injection. The data shown are mean ± SD (**P<0.01).

To investigate whether or not KLF4 also affects tumor formation ability *in vivo*, female BALB/c nude mice aged 6 to 7 weeks were used. 786-O cells infected with LV-EGFP or LV-KLF4 vectors suspended in PBS with an equal volume of Matrigel (BD Biosciences, USA) were injected into the subcutis of the armpit of nude mice. Tumor volumes were measured with calipers every 5 d after injection. The palpable tumor formation of LV-EGFP and LV-KLF4 cells occurred approximately at the same time after inoculation. However, the tumor development with LV-KLF4 cells was significantly slower than that with LV-EGFP (Figure 5 A, B). The mean volumes of the tumors that were obtained from the LV-KLF4 group (135±10 mm^3^) were significantly lower than those from the LV-EGFP group (255±20 mm^3^; *P*<0.05) when the tumors were removed from the sacrificed mice on day 45 after injection (Figures 5B and C). In addition, the averages of tumor weights derived from the LV-KLF4 group were significantly lower than those from the LV-EGFP group when the nude mice were sacrificed (Figure 5 D; *P*<0.01).

## Materials and Methods

### Ethics Statement

Written informed consent was obtained from the patients before the surgery. This study was approved by the Protection of Human Subjects Committee of the Chinese People’s Liberation Army (PLA) General Hospital.

The experimental protocols and animal care were evaluated and approved by the Animal Care and Use Committee of the First Affiliated Hospital of the Chinese PLA General Hospital. The female BALB/c nude mice were housed in cages and given food and water ad libitum. Surgeries were performed under sodium pentobarbital anesthesia and efforts were made to minimize suffering.

### Collection of ccRCC Specimens

All of the tumor samples and adjacent normal tissues were collected from surgically removed specimens at the Chinese PLA General Hospital. The samples were snap frozen in liquid nitrogen, and then stored at –80°C until analysis. The case-matched normal tissues were at least 5 cm away from the edge of the corresponding tumors. All of the RCC cases were clinically and pathologically confirmed as clear cell carcinoma and were staged based on the 2009 Union for International Cancer Control TNM (7th edition) classification of malignant tumors. The medical ethics committee of the Chinese PLA General Hospital approved this study.

### Cell Culture and Reagents

ccRCC cell lines 786-O, 769-P, Caki-2, Caki-1, and ACHN as well as normal renal proximal tubular epithelial cell line HKC were maintained in our laboratory. HKC and Caki-2, 786-O and 769-P, caki-1, and ACHN were cultured in Dulbecco’s modified Eagle’s medium (HyClone), RPMI 1640 medium (HyClone), McCoy 5A medium (HyClone), and MEM-BESS (HyClone), respectively. All of the media were supplemented with 10% fetal bovine serum (Gibco). All of the cells were cultured in a sterile incubator at 37°C with 5% CO_2_.

### RNA Isolation and Real-time PCR

RNA isolation and real-time PCR methods were performed as described previously [33]. The relative expression levels were normalized to the expression of human peptidylprolyl isomerase A (PPIA) mRNA. Relative quantification was performed using the ΔΔCt method. The KLF4 primers used for the real-time PCR analysis were as follows: forward ACAAAGAGTTCCCATCTCAA and reverse GTAGTGCCTGGTCAGTTC. We used the validated sequence for p21*^WAF1/CIP1^*, p27*^Kip1^* [33], and cyclin D1 [34].

### Western Blot Analysis

Western blot analysis was performed using standard techniques [35], in which the lysates were obtained from tissues and cells. The rabbit polyclonal antibody against KLF4 (1∶500 dilution; Sc-20691; Santa Cruz) was incubated at 4°C overnight. After the sample was washed four times with Tris-buffered saline supplemented with 0.1% Tween 20 for 7 min, the membrane was incubated with peroxidase-conjugated goat anti-rabbit IgG antibodies (1∶2000, Santa Cruz) for 1 h at room temperature. Protein bands were detected by a chemiluminescence kit (Pierce) using medical X-ray films and quantified by Photoshop (Adobe software). KLF4 expressions in each sample were normalized with β-actin or glyceraldehyde-3-phosphate dehydrogenase levels.

### Immunohistochemistry

All of the samples were fixed in 10% neutral formalin. Sections (4 µm thick) were cut from wax blocks that were mounted on APES-coated glass slides. A standard immunostaining procedure was then performed using a primary polyclonal goat antibody against human KLF4 (15 µg/mL dilution, R&D AF3640). Normal human gastric and intestinal epithelial cells were used as a positive control sample. PBS, instead of a primary antibody, was used as a negative control treatment. Protein expression was graded on a scale of “+” to “+++” as previously described [36], in which “+” indicates ≤25% or absence of positive cells.

### Methylation-specific PCR (MSP)

Genomic DNA from ccRCC tissue specimens were modified by sodium bisulfite treatment as described previously [37]. A typical CpG island that spans the proximal promoter and exon 1 regions of the KLF4 gene was found (http://cpgislands.usc.edu/). MSP primers for KLF4 were designed and synthesized (Invitrogen) according to the genomic sequences. To detect the unmethylated DNA (U), the forward primer and the reverse primer used were 5′-GTTGGTATTGTTATTGGTATTGGTG-3′ and 5′-ACACTATCTACACACTAAAAAAAAAC-3′, respectively, which covered a region of 128 bp from –581 bp to –453 bp relative to the translation initiation site of the human KLF4 exon 1 region. To detect the methylated DNA (M), the forward primer and the reverse primer were 5′-GGTATCGTTATCGGTATTGGC-3′ and 5′-CTATCTACGCGCTAAAAAAAAACG-3′, respectively, which spanned a region of 125 bp from –580 bp to –455 bp relative to the translation initiation site of the human KLF4 exon 1 region. Normal blood lymphocyte DNA, in vitro methylated DNA, and water were used as the negative control sample, the positive control sample, and the blank sample, respectively. Each MSP reaction incorporated approximately 100 ng of bisulfite-treated DNA, 25 pmol of each primer, 100 pmol of dNTPs, 2.5 µl of 10× PCR buffer, and 1unit of Taq polymerase (Invitrogen) in a final reaction volume of 25 µl. Each PCR reaction was started at 95°C for 5 min, amplified for 35 cycles (95°C for 30 s, 60°C for 30 s, and 72°C for 30 s), and subjected to a final elongation at 72°C for 5 min. The MSP products were analyzed using 2.0% agarose gel stained with ethidium bromide.

### Construction of *Lentivirus* (LV) for KLF4 Overexpression

The lv-ef1a-klf4-IRES-EGFP plasmid was kindly provided by Prof. Hai-liang Feng (Cell resource Center, Institute of Basic Medicine Sciences, Chinese Academy of Medical Sciences, China). A control vector (lv-ef1a-IRES-EGFP) was created by removing the KLF4 gene from the lv-ef1a-klf4-IRES-EGFP sequence. All of the plasmids were verified by restriction enzyme analysis and DNA sequencing. lv-ef1a-klf4-IRES-EGFP or lv-ef1a-IRES-EGFP was co-transfected with Δ8.91 and pVSV-G into 293T cells, respectively, by using lipofectamine 2000. The viral supernatant of lv-ef1a-klf4-IRES-EGFP and lv-ef1a-IRES-EGFP was filtered through a 0.45 µm filter and renamed as LV-KLF4 and LV-EGFP, respectively. All of the supernatants were used to infect the target cells with 10 µg/ml of polybrene (Sigma-Aldrich). After the target cells were infected for 96 h, KLF4 expression was confirmed by real-time PCR and western blot analyses (Figure 3B). The cells infected with LV-KLF4 and LV-EGFP were used for further analysis.

### RNAi Knockdown

Three small interfering RNA (siRNA) duplexes targeting different coding regions of human KLF4 and their scrambled sequence siRNA (mock) were custom synthesized by Shanghai Gene-Pharma Co. (Shanghai, China). For the RNAi knockdown, equal numbers of cells were seeded in the plates containing medium without antibiotics for 24 h prior to the transfection. The siRNAs were introduced into the cells using Lipofectamine 2000 in serum-free Opti-MEM, according to the manufacturer’s instructions. The expression levels of KLF4 were determined by real-time PCR and western blot analyses (Figure 3B). The most efficient siRNA for knockdown was renamed as si-KLF4 and chosen for further experiments, the scrambled sequence siRNA was renamed as si-NC. The transfected cells were grown in complete medium at 37°C and 5% CO_2._ The cells were harvested at the indicated time points and used for further analysis.

### Anchorage-dependent and Independent Growth Assays

For the anchorage-dependent growth assay, 786-O and ACHN cells were separately seeded in six-well culture plates after the cells were infected with LV-EGFP or LV-KLF4 for 96 h at a density of 1×10^3^ cells per well. Colony numbers were counted after they were fixed with 75% ethanol for 15 min and stained with 0.2% crystal violet at 14 d. Anchorage-dependent growth assay was also performed using HKC cells after transfected with si-NC or si-KLF4 for 48 h.

For the anchorage-independent growth assay, the cells in the single-cell suspension were plated in 0.35% agarose on a 0.7% agarose bottom layer at a density of 1×10^3^ cells per well in six-well plates. Afterward, the cells were incubated. Colonies were scored after three to fourweeks of growth.

### Cell Growth Analysis

MTS was used to assess cell proliferation. A total of 1×10^3^ cells were seeded into 96-well plates in 100 µl of 10% FBS/medium and incubated at 37°C in 5% CO_2_. After 24, 48, 72, 96, and 120 h, 20 ml of CellTiter 96 Aqueous One Solution (Promega) was added to each well, and then incubated for 1 h at 37°C in 5% CO_2_. Absorbance was determined at 490 nm by using a microplate reader. Each experiment was performed in triplicate and repeated thrice. Transwell invasion and migration assays (for details see File S1 ),together with wound-healing assay (for details see File S2) were also performed using 786-O cells to investigate the role of KLF4 in metastasis.

### Cell Cycle Analysis by Flow Cytometry

After 96 h of infection with LV-EGFP or LV-KLF4, the cells were collected, fixed in 70% ethanol for 30 min, and washed thrice with ice-cold PBS. Cell pellets were re-suspended in RNase-containing (1∶100 in dilution) PBS buffer in an ice bath and stained with propidium iodide (BD Biosciences, San Jose, CA) according to the manufacturer’s protocol. The stained cells were analyzed on the FACS-Calibur (BD Biosciences). Data were analyzed using the Cellquest Pro software (BD Biosciences). HKC cells after transfected with si-NC or si-KLF4 for 48 h were also used for cell cycle analysis using the same method.

### Experimental Animal

Female BALB/c nude mice (aged 6 to 7 weeks; *n* = 10) were purchased from the Academy of Military Medical Sciences (Beijing, P. R. China). After infected with LV-KLF4 or LV-EGFP for 96 h, 786-O cells were harvested, respectively. Afterward, 1×10^7^ viable cells suspended in PBS mixed with an equal volume of Matrigel (BD Biosciences USA) were injected into the subcutis of the armpit of nude mice [38]. The tumor volume (*V*) was determined according to the following equation: *V* = ab^2^/2, where a and b is the length and the width, respectively [39].

### Statistical Analysis

The results were represented as the average from triplicate experiments and expressed as the mean±SD. The associations between categorical variables were assessed using the chi-square test or the Fisher’s exact test. ANOVA was performed to determine the statistical significance among the groups. *P* value >0.05 was considered statistically significant.

## Discussion

KLF4 functions either as a tumor suppressor or an oncogene depending on different cellular contexts. Furthermore, the recently new findings reveal more controversial role of KLF4 in tumorigenesis in one single tissue, such as the newly reported potential oncogenic role of KLF4 or KLF4 isoforms in pancreatic cancer [40], colon cancer [41] and prostate cancer [42] development. However, the function and detail mechanisms of KLF4 in ccRCC development and progression remains unknown. The present study was the first to investigate the expression and potential tumor suppressive role of KLF4 in human ccRCC pathogenesis. We found that KLF4 expression was lower in ccRCC tissues than in patient-matched normal tissues both at protein and mRNA levels. Patients with weakly or negatively stained KLF4 had larger tumor size and higher tumor stage.

The loss of KLF4 expression contributed to carcinogenesis in several cancer types. Several possible causes may be accounted for the low KLF4 expression. Increasing evidence has suggested that aberrant DNA methylation of CpG islands around the promoter regions can affect the inactivation of KLF4. Hypermethylation is observed in the promoter region of KLF4 in several cancers, including colorectal cancer, hepatic cancer, and lung cancer [17], [19], [22]. Studies have also shown that the reduced or undetected KLF4 expression may be caused by the loss of heterozygosity (LOH) on chromosome 9q31 (where KLF4 is located) [43], [44]. Thus, LOH and hypermethylation may contribute to the KLF4 downregulation. Our results indicated that the reduced KLF4 expression was partly (44%) associated with the hypermethylation in its promoter region in primary ccRCC. This finding is consistent with that in previous reports, in which the methylated allele is observed in hepatic cancer [19] and primary lung cancer [22]. Although some other causes, such as gene deletion or gene mutation, may lead to the altered KLF4 expression in ccRCC, our results partially indicated that the hypermethylation in the promoter region contributed to the reduced KLF4 expression at the transcriptional level.

To investigate further the function of KLF4 in ccRCC tumorigenesis, MTS as well as anchorage-dependent and independent growth assays were performed. The ectopic KLF4 expression significantly inhibited the proliferation of ccRCC cell lines, on the contrary, knockdown of KLF4 promoted cell proliferation in normal renal proximal tubular epithelial cell line (HKC). The anti-proliferative effect of KLF4 was confirmed *in vivo.* These results provided evidence that KLF4 may function as a tumor suppressor in ccRCC carcinogenesis.

Although the detailed mechanisms by which KLF4 influences cancer development and progression remain unclear, evidence has suggested that the altered KLF4 expression affects G1/S or G2/M phase of the cell cycle [9], [18], [22]. In the present study, we found that the restoration of KLF4 expression inhibited cell proliferation by blocking the G1/S phase transition in ccRCC cell lines. Furthermore, the restoration of KLF4 upregulated the expression of p21*^WAF1/CIP1^*, which is a well-characterized CDK inhibitor. A high level of p21*^WAF1/CIP1^* inhibits cyclin D1 expression and binds to cyclin/CDK complexes [12]. p21*^WAF1/CIP1^* is also an important negative regulator of the G1/S phase transition and a major checkpoint for cell cycle progression [31], [32], [45]. After KLF4 was restored, we observed a suppressed expression of cyclin D1, another critical target of proliferative signals at the G1 phase [46], in which the promoter can also be suppressed by KLF4 via an SP1 motif [15]. The ectopic KLF4 expression up regulated the p21*^WAF1/CIP1^* expression and downregulated the cyclin D1 expression. This result provided evidence that KLF4 could change the expression of molecules, which are important in controlling the cell cycle progression. However, significant changes were not observed in the p27*^KIP1^* expression in 786-O and ACHN cells after KLF4 was overexpressed. Furthermore, converse result can be observed after KLF4 knockdown in HKC cells. This result suggested that KLF4-induced growth suppression and G1-phase arrest may be caused by inducing p21*^WAF1/CIP1^* and suppressing the cyclin D1 expression. p21*^WAF1/CIP1^* can be upregulated by a p53-independent or a p53-dependent mechanism [47]–[49]. KLF4 exhibits opposite effects on p21*^WAF1/CIP1^* and p53 in mouse embryonic fibroblasts as it suppresses p53 but induces p21*^WAF1/CIP1^* expression [50]. Although the underlying mechanism in ccRCC cells requires further investigation, the results provided evidence that the decreased KLF4 expression could enhance the cell growth via a G1/S phase arrest in ccRCC.

Kidney cancer is resistant to chemotherapy and conventional immunotherapy. New targeted drugs (NTDs), such as sunitinib and sorafenib, are the standard medications for patients with advanced or metastatic ccRCCs. However, these treatments rarely produce complete responses and curative effects are not observed [51]. For this reason, appropriate biomarkers and therapeutic targets of ccRCC are still needed. Some important oncogenes, such as p21*^WAF1/CIP1^*, p*^27Kip1^*, cyclin D1, and β-catenin, have important functions in human ccRCC development and treatment [52]–[54]. Furthermore, KLF4 can modulate these genes by binding directly to the promoter of the corresponding sequence [12]–[15]. p21*^WAF1/CIP1^*, which is upregulated in most RCCs, is a prognostic marker for RCC [55]. The increased p21*^WAF1/CIP1^* expression is related to poor prognosis of NTDs [56]. Studies have also demonstrated that β-catenin protein expression in RCC tumors is significantly greater than that in the normal kidney tissues [57]. These results indicated that the aforementioned genes have important functions in carcinogenesis and ccRCC treatment. In the present study, we found that p21*^WAF1/CIP1^* and cyclin D1 may function in ccRCC development via the KLF4 pathway.

Given the anti-proliferative effect of KLF4 on ccRCC, the restoration of KLF4 can also inhibit the migration, invasion and motilities of 786-O cells. (Figure S1, Figure S2). Moreover, cells revealed an epithelial morphology (data not shown) after KLF4 was restored in ACHN, a cell line derived from the distant metastatic ccRCC. Studies have shown that KLF4 inhibits epithelial-to-mesenchymal transition by regulating E-cadherin and slug expressions [58], [59]. Induced KLF4 expression significantly upregulated the E-cadherin expression, but not snail-1 and slug, at the transcriptional level (data not shown). Thus, the function of KLF4 in ccRCC development and progression as well as the effect of KLF4 on the metastasis of ccRCC should be further investigated.

In summary, our data showed that KLF4 functioned as a tumor suppressor in ccRCC pathogenesis. KLF4 also exhibited anti-proliferative effects on ccRCC cells *in vitro* and *in vivo*. This result suggested that KLF4 could be a potential biomarker and therapeutic target of ccRCC.

## Supporting Information

Figure S1 **KLF4 overexpression inhibited 786-O cell migration and invasion.** (A) Representative photographs were taken at 20× magnifications (left panel). The number of migrated cells was quantified in four random images from each treatment group. Results are the mean ± SD from two independent experiments (right panel) (**P<0.01). (B) Representative photographs were taken at 40× magnifications (left panel). The number of invading cells was quantified (right panel) (**P<0.01).
(TIF)

Figure S2 **KLF4 overexpression inhibited the motility of 786-O cell.** (A) Motilities of 786-O cells infected with LV-EGFP or LV-KLF4 were examined by an *in vitro* wound healing assay. Digital pictures were taken at 0 and 24 h. The black lines delineate the border of the corresponding wound.
(TIF)

File S1 **Materials and Methods, Transwell invasion and migration assay.**
(DOC)

File S2 **Materials and Methods, Wound healing assay.**
(DOC)

File S3 **Results, KLF4 inhibited ccRCC cell migration and invasion in 786-O cells.**
(DOC)
