# Supplementary figures and images for: Attenuation of Krüppel-Like Factor 4 Facilitates Carcinogenesis by Inducing G1/S Phase Arrest in Clear Cell Renal Cell Carcinoma
Source: PLoS One. 2013 Jul 5;8(7):e67758. doi: 10.1371/journal.pone.0067758 (PMC3702498; doi:10.1371/journal.pone.0067758)

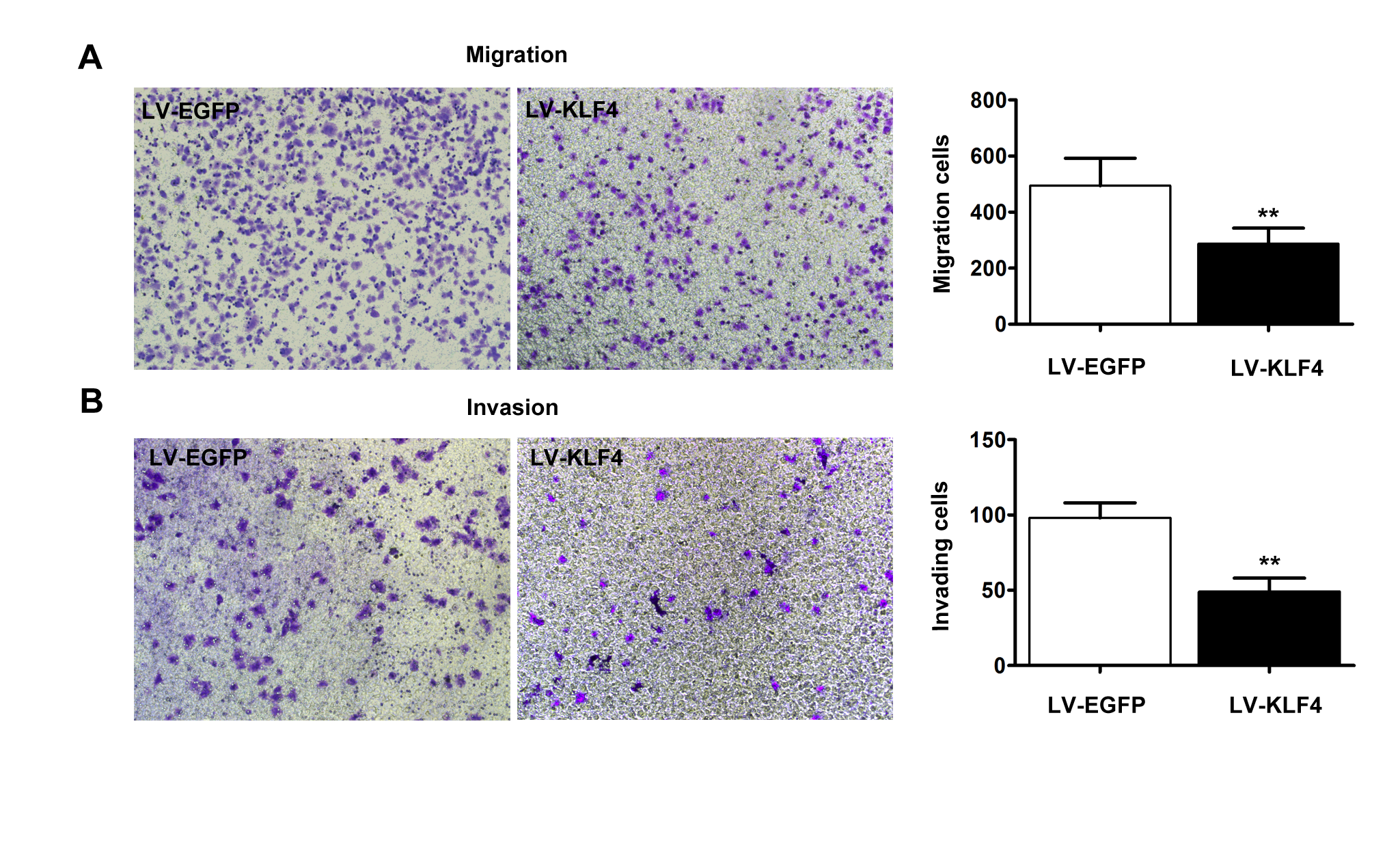

Supplement: Figure S1 — KLF4 overexpression inhibited 786-O cell migration and invasion. (A) Representative photographs were taken at 20× magnifications (left panel). The number of migrated cells was quantified in four random images from each treatment group. Results are the mean ± SD from two independent experiments (right panel) (**P<0.01). (B) Representative photographs were taken at 40× magnifications (left panel). The number of invading cells was quantified (right panel) (**P<0.01). (TIF) [file pone.0067758.s001.tif]

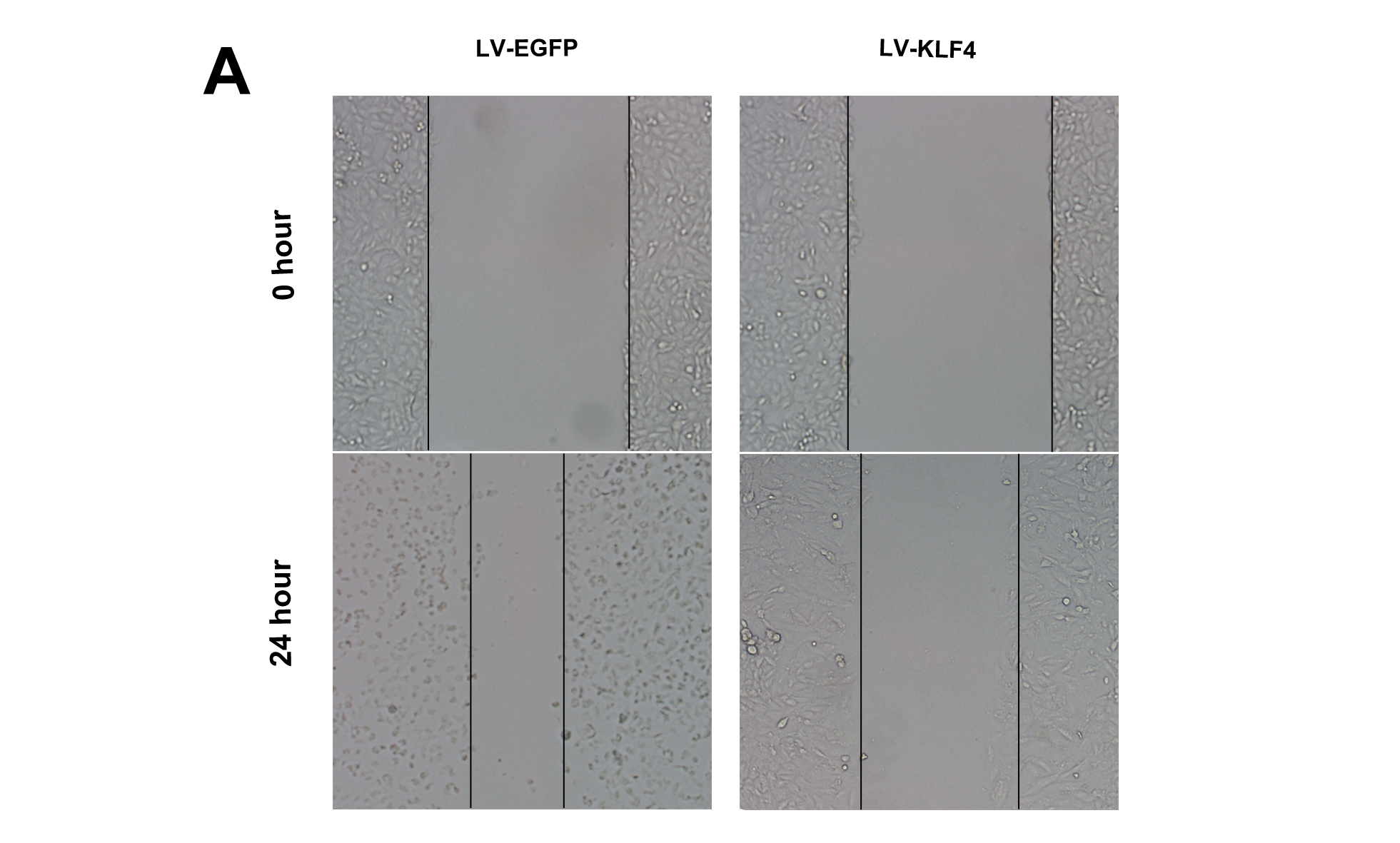

Supplement: Figure S2 — KLF4 overexpression inhibited the motility of 786-O cell. (A) Motilities of 786-O cells infected with LV-EGFP or LV-KLF4 were examined by an in vitro wound healing assay. Digital pictures were taken at 0 and 24 h. The black lines delineate the border of the corresponding wound. (TIF) [file pone.0067758.s002.tif]
