# Supplementary material for: Attenuation of Krüppel-Like Factor 4 Facilitates Carcinogenesis by Inducing G1/S Phase Arrest in Clear Cell Renal Cell Carcinoma
Source: PLoS One. 2013 Jul 5;8(7):e67758. doi: 10.1371/journal.pone.0067758 (PMC3702498; doi:10.1371/journal.pone.0067758)
Supplement: File S1 — Materials and Methods, Transwell invasion and migration assay. (DOC) [file pone.0067758.s003.doc]

**File S1**

**Materials and Methods**

**Transwell invasion and migration assay**

The invasion and migration activities of the 786-O cells infected with LV-KLF4 or LV-EGFP after 96 h were measured *in vitro*. Cells were added to the upper chamber at a density of 5 ×10^3^ cells/ 200 μl, and the lower chambers were filled with 500 μl of RPMI 1640 medium containing 10% FBS as a chemo-attractant. After incubation for 6 hours (for migration assays) or 12 hours (for invasion assays), the filter inserts were removed from the wells, and the cells on the upper side of the filter were removed using cotton swabs. The filters were fixed and stained with crystal violet. The invaded/migrated cells were then visualized and counted in five randomly selected fields (×100 magnification) under an inverted microscope. Three chambers were used for each experimental condition and each experiment was repeated thrice.
