# Supplementary material for: Attenuation of Krüppel-Like Factor 4 Facilitates Carcinogenesis by Inducing G1/S Phase Arrest in Clear Cell Renal Cell Carcinoma
Source: PLoS One. 2013 Jul 5;8(7):e67758. doi: 10.1371/journal.pone.0067758 (PMC3702498; doi:10.1371/journal.pone.0067758)
Supplement: File S2 — Materials and Methods, Wound healing assay. (DOC) [file pone.0067758.s004.doc]

**File S2**

**Materials and Methods**

**Wound healing assay**

The cells were seeded in six-well culture plates. The confluent monolayer was wounded with a plastic pipette tip, and the migration of the cells at the wound front was photographed under a microscope at indicated times after the scratch. Three independent experiments were performed.
