# Supplementary material for: Attenuation of Krüppel-Like Factor 4 Facilitates Carcinogenesis by Inducing G1/S Phase Arrest in Clear Cell Renal Cell Carcinoma
Source: PLoS One. 2013 Jul 5;8(7):e67758. doi: 10.1371/journal.pone.0067758 (PMC3702498; doi:10.1371/journal.pone.0067758)
Supplement: File S3 — Results, KLF4 inhibited ccRCC cell migration and invasion in 786-O cells. (DOC) [file pone.0067758.s005.doc]

**File S3**

**Results:**

**KLF4 inhibited ccRCC cell migration and invasion in 786-O cells**

KLF4 downregulation in tumor tissues and ccRCC cell lines suggested that KLF4 also functions in migration and invasion. Therefore, we investigated the effect of KLF4 on the cell migration and invasion of 786-O cells by using the Boyden chamber transwell assay. Figures S1 show that KLF4 overexpression inhibited the migration and invasion of 786-O cells (***P* < 0.01). For wound healing assay, overexpression of KLF4 indicated a direct inhibitive effect of motility in 786-O cells (Figures S2).
